# Dynamic Heterogeneity in Ring-Linear Polymer Blends

**DOI:** 10.3390/polym12040752

**Published:** 2020-03-30

**Authors:** Anna F. Katsarou, Alexandros J. Tsamopoulos, Dimitrios G. Tsalikis, Vlasis G. Mavrantzas

**Affiliations:** 1 Department of Chemical Engineering, Imperial College London, South Kensington Campus, London SW7 2AZ, UK; anna.katsarou96@gmail.com; 2 Division of Chemistry and Chemical Engineering, California Institute of Technology, Pasadena, CA 91125, USA; tsamoalex1996@gmail.com; 3 Department of Chemical Engineering, University of Patras and FORTH-ICE/HT, GR 26504 Patras, Greece; 4 Particle Technology Laboratory, Department of Mechanical and Process Engineering, ETH Zürich, CH-8092 Zürich, Switzerland

**Keywords:** dynamic heterogeneity, rings, threading events

## Abstract

We present results from a direct statistical analysis of long molecular dynamics (MD) trajectories for the orientational relaxation of individual ring molecules in blends with equivalent linear chains. Our analysis reveals a very broad distribution of ring relaxation times whose width increases with increasing ring/linear molecular length and increasing concentration of the blend in linear chains. Dynamic heterogeneity is also observed in the pure ring melts but to a lesser extent. The enhanced degree of dynamic heterogeneity in the blends arises from the substantial increase in the intrinsic timescales of a large subpopulation of ring molecules due to their involvement in strong threading events with a certain population of the linear chains present in the blend. Our analysis suggests that the relaxation dynamics of the rings are controlled by the different states of their threading by linear chains. Unthreaded or singly-threaded rings exhibit terminal relaxation very similar to that in their own melt, but multiply-threaded rings relax much slower due to the long lifetimes of the corresponding topological interactions. By further analyzing the MD data for ring molecule terminal relaxation in terms of the sum of simple exponential functions we have been able to quantify the characteristic relaxation times of the corresponding mechanisms contributing to ring relaxation both in their pure melts and in the blends, and their relative importance. The extra contribution due to ring-linear threadings in the blends becomes immediately apparent through such an analysis.

## 1. Introduction

Orientational relaxation in polymers is typically quantified by examining the rate of decay of the time autocorrelation function (TACF) of a unit vector directed along the longest polymer distance and computing the time needed for this function to drop to zero. For a linear polymer, this unit vector is along the direction of the chain end-to-end distance; for a ring polymer, it is along one of the diameter vectors of the molecule. In practice, the TACF curve is usually fitted to a stretched exponential (KWW) function which helps approximate the corresponding characteristic relaxation time from the integral below the curve (which can be obtained analytically). In the literature, in almost all cases, one reports the relaxation time computed from the average TACF, i.e., the function obtained by averaging over the full ensemble of chains in the system. An additional average is often performed over all possible time origins (because for the dynamics of a system at equilibrium, only time differences matter). However, in the presence of strong topological constraints, individual chains experience temporarily different local environments, hence different topological interactions; thus, it is logical to expect them to be relaxing with different rates. This is true not only in melts of well-entangled polymers but also in blends of ring-linear polymers due to strong threading events, i.e., strong penetrations of ring molecules by linear chains. The experimental investigation of this transient dynamics of individual molecules or a small fraction of their total population is not an easy task. A technique that has contributed significantly to addressing such a problem over the years is fluorescence microscopy (FM) combined with single-molecule tracking techniques. 

Already since 1994, the Chu group employed FM to visualize for the first time linear chain retraction inside a “tube” [1]. In a later study from the same group [2], it was found that long linear DNA molecules in dilute aqueous solution under elongational flow exhibit an unusual form of molecular individualism [3]; DNA molecules of identical size and deformation history were found to exhibit distinct conformational shapes and dynamics that were strongly heterogeneous [2]. Almost a decade later, Teixeira et al. [4] examined the dynamics and conformation of individual DNA molecules by carrying out different shear flow experiments of semidilute and entangled aqueous DNA solutions, in combination with FM. Their measurements suggested again that DNA molecules exhibit a high degree of molecular individualism. In all experiments, a broad range of conformational distributions was observed, especially at high shear rates [4]. In a more recent study, Zhou and Schroeder [5] used single-molecule FM to investigate polymer concentration effects on stress relaxation after deformation of long linear DNA molecules. With increasing polymer concentration, DNA molecules were observed to exhibit strong dynamic heterogeneity due to the formation of chain entanglements. In particular, the measurements pointed out to the existence of two subpopulations of DNA molecules in the solution: DNA molecules exhibiting a single relaxation mode and DNA molecules exhibiting a double relaxation mode. The authors suggested that the double-relaxation mode, which was found to prevail at high polymer concentrations, includes a concentration-dependent fast chain retraction mechanism followed by a slower mechanism corresponding to reptation.

Dynamic heterogeneity of ring molecules in linear matrices was investigated for the first time to our knowledge by Habuchi et al. [6]. Using single-molecule fluorescence microscopy (SMFM), these authors measured the diffusion dynamics of cyclic poly(tetrahydrofuran)s in matrices of linear counterparts. Careful analysis of their experimental measurements revealed different modes in the diffusion for rings, which were found to be related to the dynamics of individual molecules for timescales longer than the timescale of the experiment. The observed dynamic heterogeneity was attributed to topological constraints arising by partial threading of rings from linear chains [6]. A subsequent study [7] examined the diffusion and relaxation of entangled ring and linear DNA molecules at the single-molecule level, by combining FM with the cumulative area (CA) tracking technique. The diffusion coefficient of rings in the entangled matrices (*D*_C-L_) was found to be smaller than that of the linear analogs (*D*_L-L_) in the same matrix. Ring molecule diffusion was also found to be strongly heterogeneous (more than that of the linear DNA molecule). This heterogeneity was reflected on the broader distribution of *D*_C-L_ compared to *D*_L-L_, and was attributed to ring-linear threadings, suggesting that the observed broader *D*_C-L_ distribution might imply that the diffusion rate is dependent on the degree of threading (in particular, on the presence of multiply threaded rings). Using SMFM techniques, Zhou et al. [8] have also examined the relaxation dynamics and transient stretching behavior of DNA rings in semidilute unentangled linear DNA solutions. They found that rings undergoing extensional flow exhibit a broad distribution of conformations, even for times long after the stretching process has stopped. It was also found that ring conformational fluctuations become stronger with increasing fraction of linear chains. Unexpectedly, DNA molecules were found to exhibit large conformational variations even at tiny concentrations (*c* << c*) of linear DNA molecules in the background solution. The experimental results suggested that the observed conformational fluctuations of the ring DNA molecules are caused by the partial threading of linear DNA into open ring conformations in extensional flow.

From a simulation point of view, the individual behavior of rings in matrices of linear chains at the molecular level under equilibrium conditions has been addressed through Monte Carlo simulations by Yang et al. [9]. These authors found that short rings exhibit structural and dynamic heterogeneity due to different threading states of certain subpopulations of the ring molecules, suggesting that ring diffusion involves a combination of elements from three different mechanisms: (a) constraint release [10]; (b) restricted reptation [11]; and (c) diffusion following the contour of the linear chains [12].

By performing a direct geometric analysis of ring-linear penetrations in trajectories stored in the course of very long molecular dynamics (MD) simulations of ring-linear polyethylene oxide (PEO) blends, Tsalikis and Mavrantzas [13] found that many of the threadings are long-lived topological interactions, often remaining active for as long as 15 times the average relaxation time of rings in their own pure melt. However, their effect on the orientational relaxation of the chains suffering threadings was not systematically examined. It is exactly the purpose of the present contribution to study how differently ring molecules relax in a ring-linear blend, and in case they relax with different rates, how these differences are affected by the molecular length of the chains and the composition of the blend (relative population of ring and linear chains). To simplify the analysis, we will restrict ourselves only to symmetric blends, i.e., to blends for which linear and ring molecules are exactly of the same length. A recent topological analysis [14] revealed strong threading events between linear and ring molecules in such blends, which in many cases are multiple threadings: one ring molecule can be simultaneously threaded by many linear chains, or one linear chain can simultaneously thread many ring molecules. Our plan here is to examine the relaxation behavior of all ring molecules in the simulated ring-linear blends by monitoring the time decay of their individual TACF, fitting it with a KWW function to get a good estimate of the corresponding relaxation time, and analyzing the statistical properties of the ensemble of relaxation times collected to get information for the heterogeneous (or not) character of ring molecule relaxation.

The structure of the rest of our paper is as follows. In Section 2 we review the model ring-linear PEO blends examined and the methodology followed to address the issue of heterogeneity. In Section 3 we present our results for the orientational relaxation of the ring molecules in the simulated blends and discuss their dependence on the size of ring/linear chains and relative concentration of the blend in linear chains. Our paper ends with Section 4 presenting a quick summary of the most important findings of the work.

## 2. Systems Studied and Simulation Details

As in all our MD studies so far on the ring and linear PEO melts [15,16], the ring molecules are represented by the formula −CH_2_−O−(CH_2_−CH_2_−O)*_n_*−CH_2_− and the linear ones by the formula CH_3_−O−(CH_2_−CH_2_−O)*_n_*−CH_3_, where *n* denotes the number of monomers per molecule. We also adopt the notation that the ring component in the blend is denoted by R and the linear one by L. In the present work, we have focused on two PEO sizes characterized by *n* = 40 and 120, respectively, with corresponding molecular weights equal to 1846 and 5326 g/mol; we will refer to them as the PEO-2k and PEO-5k systems, respectively. The entanglement molecular weight *M*_e_ of linear PEO is *M*_e_ = 2020 g/mol and corresponds to *n*_e_ = 46; thus, the PEO-2k melt is unentangled while the PEO-5k one marginally entangled, characterized by Z ≈ 2.5 entanglements per chain. For each one of these systems, we simulated mixtures at molar fraction *φ*_L_ of the L component in the range between 0.1 and 0.7 (see Table 1). The MD simulations were performed with GROMACS [17] using the modified TraPPE (=Transferable Potential for Phase Equilibria) united-atom (UA) force field [18,19] for which several simulations in the last years have shown that it can predict quite accurately the equilibrium conformation and dynamics of PEO rings both in their pure melts and in blends with linear analogs judging from their comparison against experimentally measured data with state-of-the-art techniques [14,16,20].

The MD simulations were carried out in the isothermal–isobaric (NPT) statistical ensemble by making use of the Nosé-Hoover thermostat [21,22], coupled with the Parrinello-Rahman barostat [23] to constrain the temperature *T* and pressure *P* to the desired values (*T* = 363 K and *P* = 1 atm). Initial configurations were prepared with the commercially available MAPS modulus [24]. We also note that the simulation results for the pure ring melts analyzed here have already been presented in a recent contribution [25] on the study of the shear rheology of ring PEO melts in comparison to the corresponding shear rheology of their equivalent linear melts.

## 3. Results

**Figure 1:**
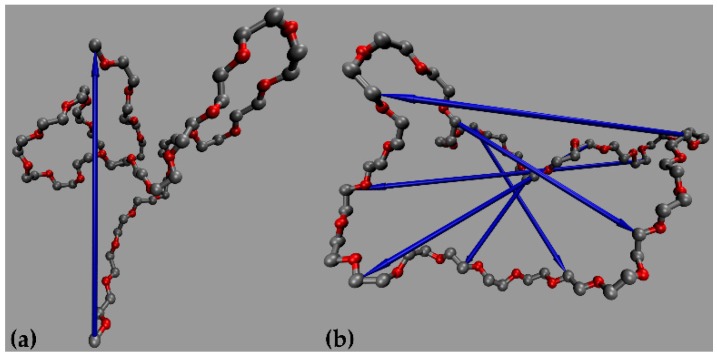
Definition of the characteristic vectors involved in the analysis of terminal orientational relaxation in linear and ring polymers. (**a**) The end-to-end vector **R**_ee_ (blue arrow) in the case of a linear PEO chain, and (**b**) various diameter vectors **R**_d_ (blue arrows) in the case of a ring PEO molecule. Grey and red beads indicate carbon and oxygen atoms along a PEO molecule, respectively.

As already discussed in the Introduction, terminal relaxation in polymers is typically analyzed by computing the decay of the TACF 〈u(t)⋅u(0)〉 of the unit vector **u** directed along the longest chain dimension. For linear chains, **u** is taken to be along the chain end-to-end vector **R**_ee_, see Figure 1a. For ring molecules, **u** is taken to be along the diameter vector **R**_d_, namely the vector between all atoms that are *N*/2 − 1 bonds apart. The results are averaged over all possible diameter vectors along a ring molecule (e.g., from atom 1 to atom *N*/2, from atom 2 to atom *N*/2 + 1, from atom 3 to atom *N*/2 + 2, …, from atom *N*/2 − 1 to atom *N*). Typical examples of such **R**_d_ vectors are shown by the blue arrows in Figure 1b. The rate with which 〈u(t)⋅u(0)〉 approaches the zero value is a measure of how fast the molecule forgets its initial configuration, thus also a measure of its overall orientational relaxation.

**Figure 2:**
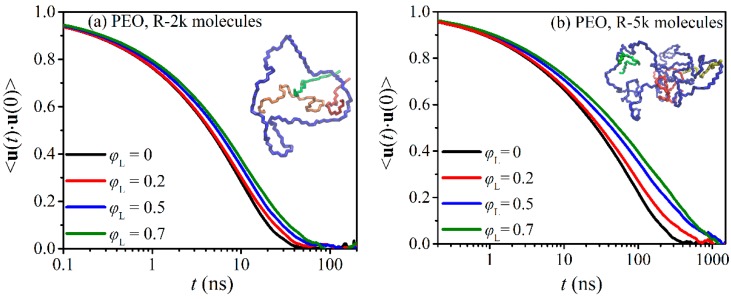
Decay of the time autocorrelation function 〈u(t)⋅u(0)〉 averaged over all ring molecules in: (**a**) the PEO-2k blends, and (**b**) the PEO-5k blends. The curves for the two pure ring melts are from Ref. [25].

Then, to describe terminal relaxation we resort to a description of the 〈u(t)⋅u(0)〉 curves through a KWW function of the form:

(1) $$\langle u\left(t\right)\cdot u\left(0\right)\rangle =\exp\left[-{\left(t/\tau_{KWW}\right)}^{\beta }\right],$$

where $\tau_{KWW}$ and *β* denote the characteristic KWW time and stretching exponent, respectively. Typical simulation results for the function 〈u(t)⋅u(0)〉 for the ring molecules in the simulated PEO-2k and PEO-5k blends are shown in Figure 2. We observe that the presence of linear chains causes an appreciable shift in the TACF curves to the right; with increasing *φ*_L_, the function 〈u(t)⋅u(0)〉 needs more time to drop to zero. The effect becomes more pronounced with increasing chain length of rings (and linears). For example, in the pure R-5k melt, the function 〈u(t)⋅u(0)〉 drops to zero after ~560 ns while in the blend with *φ*_L_ = 0.5 the corresponding time is ~1100 ns (i.e., it doubles).

To quantify the effect of the presence of the linear chains on the orientation dynamics of the rings, we computed the so-called (orientational) relaxation time $\tau_{0}$ defined from the integral below the 〈u(t)⋅u(0)〉-vs.-*t* curve, which is mathematically given by

(2) $$\tau_{0}=\tau_{KWW}\Gamma \left(1/\beta \right)/\beta ,$$

where Γ denotes the gamma function. The values of the set $\left\{\tau_{ΚWW},\beta ,\tau_{0}\right\}$ of the three characteristic constants for all blends simulated are reported in Table 2. For the stretching exponent *β*, values between 0 and 1 (*β* = 1 corresponds to the exponential function) are expected. The smaller the value of *β* from 1 the higher the degree of dynamic heterogeneity in the melt [26]. We observe that in all blends simulated here, the value of *β* is significantly smaller than its value in the corresponding pure ring melt, a direct indication that ring molecule dynamics in the presence of linear chains are more heterogeneous. The effect is more pronounced in the case of the PEO-5k blends, for which our simulations indicate rather small *β* values. For example, the value of *β* in the PEO-2k and PEO-5k blends with *φ*_L_ = 0.2 is smaller by ~9% and ~12%, respectively, compared to its value in the respective pure melt at the same temperature. An interesting observation is that by further increasing the concentration of linear chains to *φ*_L_ = 0.5, the value of *β* remains practically unaffected in the PEO-2k blend, whereas in the PEO-5k a reduction of ~25% is observed compared to the pure ring analog (R-5k melt). Increasing the concentration of linear chains even further in the two types of blends does not seem to affect the value of *β* anymore.

As far as the terminal relaxation time $\tau_{0}$ is concerned, our simulation data in Table 2 show that its value increases rapidly with increasing fraction of the linear component, implying a strong slowing down of ring dynamics. For example, compared to its value in the corresponding pure ring melt, in the blends with *φ*_L_ = 0.5, $\tau_{0}$ is by 38% higher in the case of the PEO-2k and by 160% in the case of the PEO-5k. Overall, the longer the size of the rings or the higher the concentration of the blend in linear chains, the larger the slow-down of ring relaxation and the stronger the dynamic heterogeneity. As we have shown in a recent publication [14], ring relaxation in the blends is dramatically affected by the intensity of regular or transient threading events that ring molecules undergo with the linear chains present in the melt, and how fast these are created or released [13]. The terminal relaxation time $\tau_{0}$ in the R-2k, R-5k and R-10k blends containing linear analogs at *φ*_L_ = 0.9 (at *T* = 413 K) increased [14] by ~166%, ~420% and ~550%, respectively, compared to its value in the corresponding pure melt. Due to their more open conformations, longer rings experience more threadings by linears than shorter rings, which in addition live for longer times. This causes a dramatic reduction in their diffusion and relaxation dynamics [6,9,27], which in turn enhances dynamic heterogeneity in the blend, as many of these topological interactions are multiple threadings characterized by a wide spectrum of lifetimes [13].

**Figure 3:**
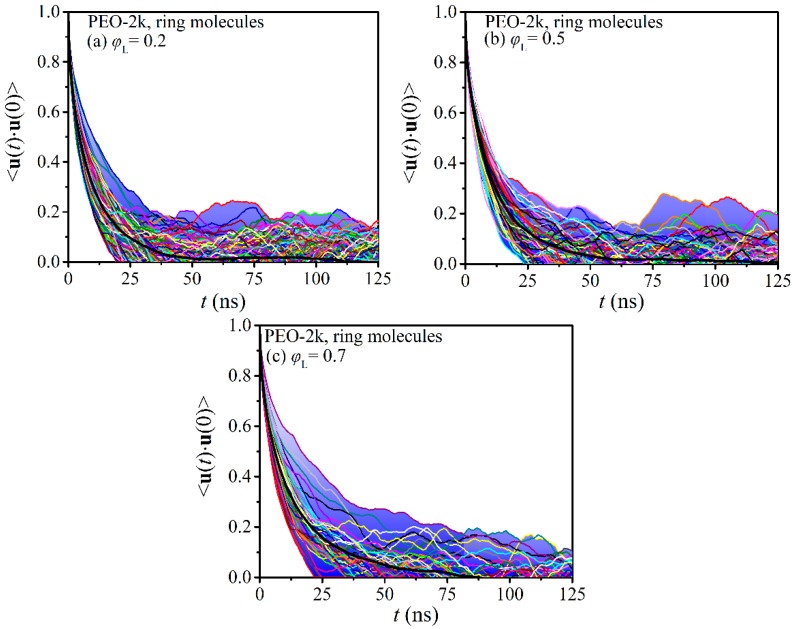
Decay of the time autocorrelation function 〈u(t)⋅u(0)〉 for each ring PEO molecule separately in the simulated ring-linear PEO-2k blends at different concentrations of the linear component: (**a**) *φ*_L_ = 0.2, (**b**) *φ*_L_ = 0.5, and (**c**) *φ*_L_ = 0.7.

**Figure 4:**
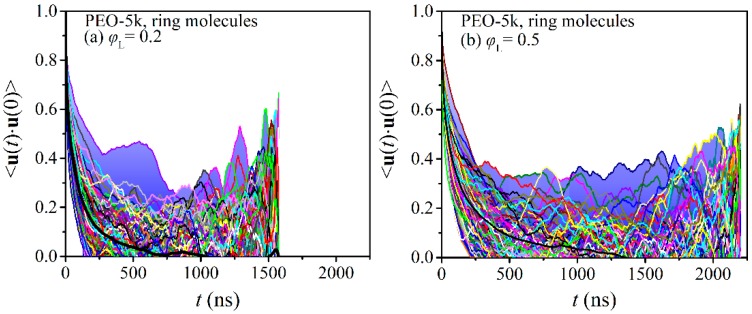

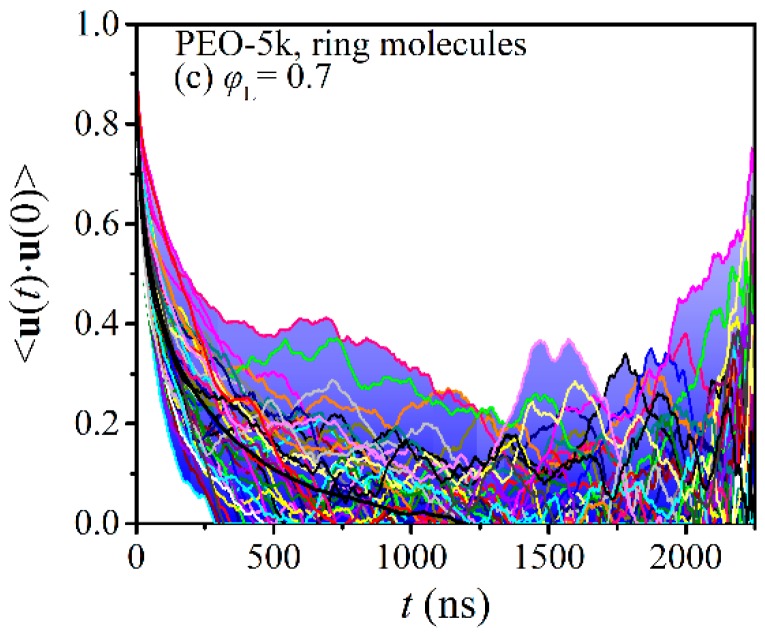
Decay of the time autocorrelation function 〈u(t)⋅u(0)〉 for each ring PEO molecule separately in the simulated ring-linear PEO-5k blends at different concentrations of the linear component: (**a**) *φ*_L_ = 0.2, (**b**) *φ*_L_ = 0.5, and (**c**) *φ*_L_ = 0.7.

To shed additional light on the effect of molecular length and blend composition on the issue of dynamic heterogeneity in ring-linear blends, we calculated the TACF curves of all ring molecules in the two melts (PEO-2k and PEO-5k). In all cases, long MD simulations were performed to ensure that the individual TACFs for all ring molecules had dropped to zero. The results are collectively shown in Figure 3 and Figure 4, together with the average curve (thick black line) in each case. With blue, we have marked the area below those TACF curves that were found to deviate the most from the average 〈u(t)⋅u(0)〉 curve.

Focusing first on the PEO-2k blends, we observe that with increasing concentration in linear chains, the curves of the individual TACFs deviate more from the corresponding average dynamics. The blue-shaded areas span a broader range of 〈u(t)⋅u(0)〉 values as the population of linear chains increases. With increasing molecular weight, the degree of dynamic heterogeneity is enhanced. To fully relax all rings in the blends, simulations on the order of a few microseconds were required, especially at the larger *φ*_L_ values. We also see that the individual ring TACFs span a broader range of 〈u(t)⋅u(0)〉 values with increasing *φ*_L_.

**Figure 5:**
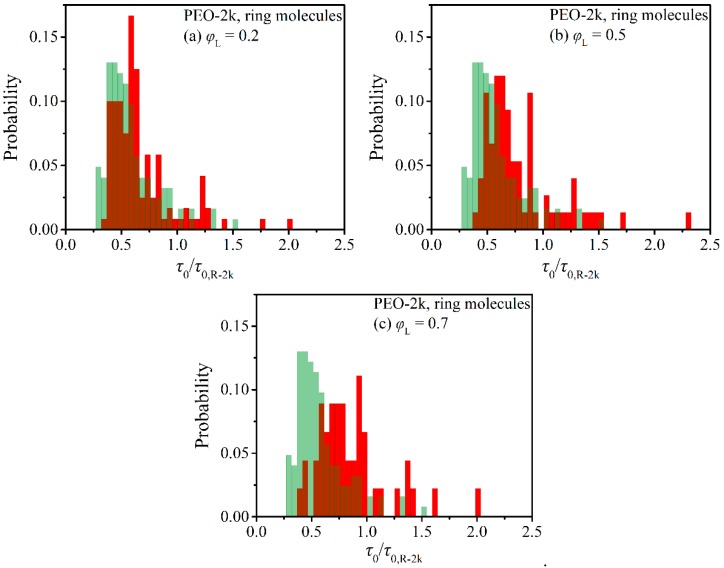
Histograms (red bars) of the characteristic ring orientational relaxation times in the simulated PEO-2k blends as a function of the fraction of linear chains: (**a**) *φ*_L_ = 0.2, (**b**) *φ*_L_ = 0.5, (**c**) *φ*_L_ = 0.7. Green bars represent the corresponding histograms in the pure ring melt from Ref. [25].

**Figure 6:**
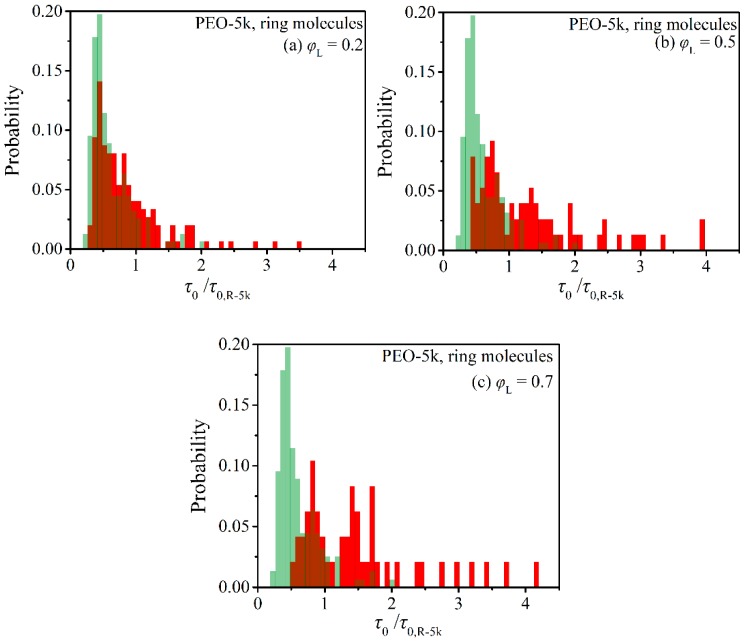
Histograms (red bars) of the characteristic ring orientational relaxation times in the simulated PEO-5k blends as a function of the fraction of linear chains: (**a**) *φ*_L_ = 0.2, (**b**) *φ*_L_ = 0.5, (**c**) *φ*_L_ = 0.7. Green bars represent the corresponding histograms in the pure ring melt from Ref. [25].

From each individual ring TACF, the corresponding relaxation time was estimated from the integral below the curve from the initial time *t* = 0 up to the final time *t* = *t*_max_ where the TACF was observed to cross over to negative values for the first time. From the ensemble of the collected individual ring relaxation times, we computed next their histograms, and the results are displayed in Figure 5 and Figure 6. To enable a direct comparison between the two blends (PEO-2k and PEO-5k) and to reveal the role of molecular size on dynamic heterogeneity, the individual ring relaxation times are shown normalized with the corresponding average ring relaxation time in the respective pure ring melt.

According to Figure 5a, the presence of linear chains in the PEO-2k blends causes a small but appreciable change in the distribution of relaxation times $\tau_{0}$ compared to the corresponding picture in the pure R-2k melt. Even if the shape of the distributions is the same and their maxima are observed approximately at the same time (at around $\tau_{0}/\tau_{0,\text{R-}2k}$ = 0.6), the presence of linear chains in the blends delays significantly the relaxation of a good subpopulation of rings. We understand that this is linked to strong threadings of these rings with some of the linear chains. Threaded ring molecules need considerably much longer time to relax than unthreaded ones, which pushes the tail of the distribution to longer times ($\tau_{0}/\tau_{0,\text{R-}2k}$ ≥ 1). However, due to their small size, linear PEO-2k chains cannot thread many rings. A rule of thumb implied by experimental findings and supported by molecular simulations [13] is that a linear chain cannot thread more rings than its number of entanglements. Therefore, the largest population of rings in the PEO-2k blend at *φ*_L_ = 0.2 remain unthreaded, thus the histogram of their characteristic relaxation times resembles the corresponding histogram of the pure R-2k melt. However, by further increasing the concentration of linear chains (see Figure 5b,c), the distribution of times broadens up considerably, with the percentage of rings characterized by relaxation times $\tau_{0}/\tau_{0,\text{R-}2k}$ > 1 increasing from ~5% in the pure ring melt to ~12% in the blend with *φ*_L_ = 0.2, to ~18% in the blend with *φ*_L_ = 0.5, and finally to ~20% in the blend with *φ*_L_ = 0.7. The same behavior is observed for the median of the distribution, which is pushed to larger and larger times as the relative population of linear chains increases. For example, the time needed for 50 % of the rings for full orientational relaxation in our simulations increases from $\tau_{0}/\tau_{0,\text{R-}2k}$ = 0.5 in the pure ring R-2k melt to $\tau_{0}/\tau_{0,\text{R-}2k}$ = 0.57 in the blend with *φ*_L_ = 0.2, to $\tau_{0}/\tau_{0,\text{R-}2k}$ = 0.66 in the blend with *φ*_L_ = 0.5, and eventually to $\tau_{0}/\tau_{0,\text{R-}2k}$ = 0.77 in the blend with *φ*_L_ = 0.7. As already mentioned several times before, this dramatic increase is the direct consequence of weak or strong threadings of rings by linear chains. Our results are in line with the work of Tsalikis and Mavrantzas [13] according to which, in unentangled ring-linear blends with *φ*_L_ = 0.75, the majority of rings are threaded by only one linear chain. From the remaining, a small subpopulation is multiply-threaded but there also exists a good percentage (close to 35%) that are completely free (not threaded at all).

As the molecular length of the ring and linear molecules in the blend increases from 2k to 5k (see Figure 6), the observed features of dynamic heterogeneity become more pronounced despite the fact that the shape of the histograms remains again unaltered (e.g., in both the 2k and 5k blends with *φ*_L_ = 0.2, the maximum is observed at the same time, around $\tau_{0}/\tau_{0,\text{R-}2k}$ = 0.5). Particularly striking is the emergence of a small subpopulation of ring molecules in the 5k blend at *φ*_L_ = 0.2, which is characterized by significantly larger relaxation times than in the corresponding 2k blends. Interestingly enough, by further increasing the concentration of linear chains in the blend, this maximum disappears, the distribution becomes more uniform, and the subpopulation of rings characterized by long relaxation times increases considerably. For example, the percentage of rings that are characterized by relaxation times longer than $\tau_{0}/\tau_{0,\text{R-}5k}$ = 1 is ~7% in the case of the pure R-5k melt, but this increases to ~25% in the blend with *φ*_L_ = 0.2, to ~52% in the blend with *φ*_L_ = 0.5, and finally to ~62% in the blend with *φ*_L_ = 0.7. The strong impact of the presence of linear chains on the relaxation dynamics of rings in the case of the PEO-5k blends is additionally reflected in the shift of the median of the distribution to higher values.

**Figure 7:**
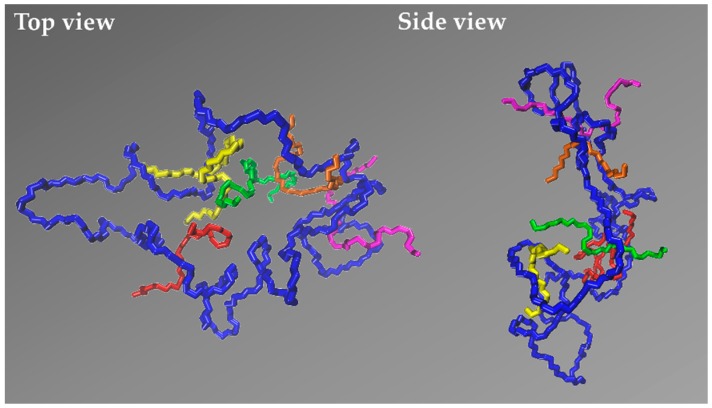
Atomistic snapshot of a multiply threaded ring from the MD simulation with the PEO-5k blend at *φ*_L_ = 0.5. The blue ring is simultaneously threaded by five linear chains (shown in red, yellow, green, orange, and magenta). For simplicity, only the parts of the linear chains involved in the threading are shown.

Overall, our simulations reveal that the dynamic heterogeneity in the simulated ring-linear PEO blends is strongly linked to the size of the two components, also to their relative population. The rather long linear chains in the 5k blends can thread more rings than the shorter linear chains in the 2k blends; and similarly, the larger PEO-5k rings experience more penetrations by linear chains than the smaller PEO-2k rings. Moreover, increasing the relative population of linear chains in the longer PEO-5k blends increases the probability to observe multiply threaded rings. As has been discussed by Tsalikis and Mavrantzas [13], in the marginally entangled PEO-5k ring-linear blend at *φ*_L_ = 0.75, more than 90% of the rings are threaded by linear chains and more than ~45% of the threaded rings are multiply threaded. It is these multiply threaded rings that are responsible for the shift of the median of the distribution to higher relaxation times and the emergence of the extended tail in the regime of long relaxation times ($\tau_{0}/\tau_{0,\text{R-}5k}$ ≥ 2) in the PEO-5k blends compared to the PEO-2k ones. A characteristic example of a multiply threaded ring from our MD simulations with the 5k blend at *φ*_L_ = 0.5 is illustrated in Figure 7, with the ring and the linear PEO molecules involved in the threading depicted in their detailed atomistic representation. The blue ring is simultaneously threaded by five different linear chains (the yellow, the green, the orange, the magenta, and the red).

Relaxation times associated with different relaxation mechanisms in the simulated ring-linear PEO blends can be identified by fitting the ensemble-average TACF 〈u(t)⋅u(0)〉 for ring molecules not by the stretched exponential function of Equation (1) but by the sum of simple exponential functions with different characteristic relaxation times:

(3) $$\langle u\left(t\right)\cdot u\left(0\right)\rangle =\sum_{i=1}^{n_{m}}A_{i}\exp\left(-t/\tau_{i}\right),$$

where *n*_m_ is the total number of relaxation modes, *τ_i_* the characteristic relaxation time corresponding to the *i*-th mode, and *A_i_* a numerical constant (the pre-exponential factor). The constants *A_i_* should satisfy $\sum_{i=1}^{n_{m}}A_{i}=1$ and their magnitude is indicative of the relative importance of the corresponding relaxation mode to the overall dynamics.

**Figure 8:**
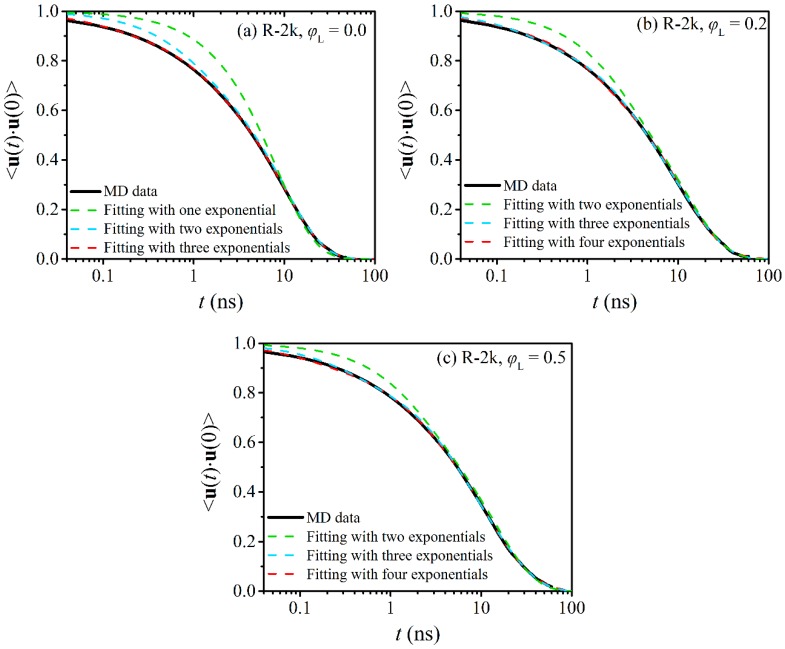
Decay of the ensemble-average TACF 〈u(t)⋅u(0)〉 describing relaxation at the level of the ring diameter vector for R-2k rings in (**a**) their pure melt, (**b**) the R-2k blend with *φ*_L_ = 0.2, and (**c**) the R-2k blend with *φ*_L_ = 0.5. The dashed lines are best fits to the MD data with Equation (3).

**Figure 9:**
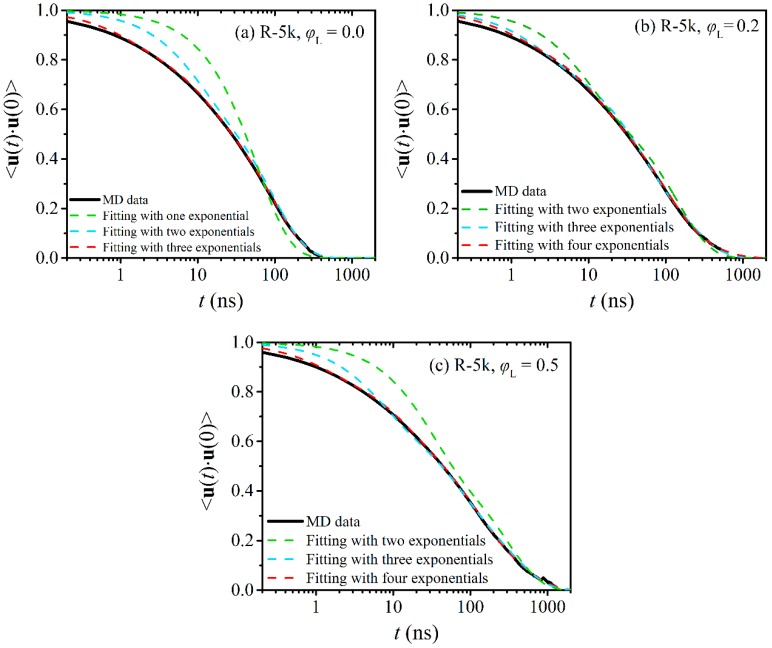
Decay of the ensemble-average TACF 〈u(t)⋅u(0)〉 describing relaxation at the level of the ring diameter vector for R-5k rings in (**a**) their pure melt, (**b**) the R-5k blend with *φ*_L_ = 0.2, and (**c**) the R-5k blend with *φ*_L_ = 0.5. The dashed lines are best fits to the MD data with Equation (3).

The fittings for different values of *n*_m_ are shown in Figure 8 for the R-2k blends and in Figure 9 for the R-5k blends. For direct comparison, we have also included in these figures the corresponding curves (MD data and numerical fits) for the two pure ring melts. From Figure 8a and Figure 9a we see that for both melts (pure R-2k and pure R-5k) the smallest total number of relaxation modes *n*_m_ needed to describe ring relaxation is *n*_m_ = 3 (i.e., we need three different exponential decay functions in Equation (3) to capture well the entire 〈u(t)⋅u(0)〉 curve). The best-fit values of the characteristic relaxation times *τ_i_* and pre-exponential factors *A_i_* for each one of the three modes are reported in Table 3. We see that for the R-2k pure melt: (a) *τ*_1_ = 0.10 ± 0.01 ns, (b) *τ*_2_ = 1.1 ± 0.1 ns, and (a) *τ*_3_ = 10 ± 1 ns, while for the R-5k pure melt: (a) *τ*_1_ = 1.1 ± 0.1 ns, (b) *τ*_2_ = 11 ± 1 ns, and (a) *τ*_3_ = 95 ± 8 ns. The longest characteristic relaxation time *τ*_3_ for both rings is on the order of the total relaxation time $\tau_{0}$ as predicted by Equation (2) and reported in Table 2. Clearly, the *i* = 3 mode is related to terminal relaxation due to translational diffusion of the entire ring molecule. We further hypothesize that the two faster relaxation modes (*i* = 1 and *i* = 2) are related to the early time motion of the loops formed by small segments along the ring molecule as, e.g., is visualized by the lattice animal picture model [28,29]). Indeed, SANS measurements [30] validated by MD simulations [20] have revealed that at short times the dynamics of rings are governed by the internal relaxation dynamics of such loops, while at intermediate time scales loop migration prevails. At times on the order of the Rouse relaxation time, these contributions get saturated, and the translational motion of the entire molecule starts dominating ring dynamics [30]. Our conjecture is therefore that *τ*_1_ and *τ*_2_ are associated with the fast loop relaxation dynamics and the slower loop migration mechanism, respectively. Atomistic simulations have also shown [20] that the saturation times for loop dynamics increase substantially with the *M*_w_ of the ring, which is fully consistent with the differences observed in the values of *τ*_1_ and *τ*_2_ between R-2k and R-5k melts. Another important remark is that the three individual characteristic relaxation times *τ*_1_, *τ*_2_ and *τ*_3_ exhibit an almost quadratic dependence on *M*_w_. This finding is in accordance with a recent simulation study [16] according to which the time $\tau_{0}$ describing terminal relaxation in pure ring melts scales with ring molecular weight as: $\tau_{0}\;\sim \;M_{w}^{1.9\pm 0.1}$.

While terminal relaxation in the two pure ring melts (R-2k and R-5k) is captured well with the sum of three exponential functions (*n*_m_ = 3), for the blends we see that we need four relaxation modes (*n*_m_ = 4) to accurately describe terminal relaxation, see Figure 8b,c for the R-2k blends and Figure 9b,c for the R-5k blends. The values of the corresponding characteristic times *τ_i_* and pre-exponential constants *A_i_* are reported in Table 3. We observe that the fourth relaxation mode is characterized by a much longer relaxation time *τ*_4_ (and a numerical constant *A*_4_ whose value increases as the percentage of linear chains in the blend increases) than the *τ*_3_ relaxation time in the corresponding pure melt. A logical inference is that this fourth relaxation mode is associated with the relaxation of that subpopulation of rings that are strongly threaded in the two types of blends (R-2k and R-5k). Such a hypothesis is further supported by the fact that the two faster characteristic times *τ*_1_ and *τ*_2_ and the corresponding numerical constants *A*_1_ and *A*_2_ remain the same as we go from the pure melts to the blends. This further seems to suggest that internal loop dynamics is a rather independent motion, not influenced at all by the degree of threading of the ring molecules by linear chains. As far as the third relaxation mode is concerned, this appears to have a weak dependence on the degree of linear contamination: the relaxation time *τ*_3_ itself is not affected by the presence of linear chains or not but the value of the pre-exponential factor *A*_3_ decreases considerably as *φ*_L_ increases. From this, we understand that the subpopulation of rings that do not experience threadings with the linear chains or is transiently threaded is reduced as the relative population of linear chains increases while their orientational relaxation remains more or less the same. The decrease in the values of *A*_3_ is more evident in the R-5k blends, since L-5k chains can thread more rings than L-2k chains. From Table 3, we also observe that the values of the two constants (the characteristic relaxation time *τ*_4_ and the pre-exponential factor *A*_4_) associated with multiple threading events in the blends depend on the fraction of linear chains present in the blend but for the precise quantification of such a dependence we need some additional simulations at some extra values of *φ*_L_. Future work could also investigate how the picture presented here changes in ring-linear PEO blends of even longer molecular weight such as e.g., the R-10k blends already simulated in Ref. [14]. A statistical analysis of dynamic heterogeneity in these blends was not pursued here because of the small number of rings present in the simulation cells of Ref. [14] for the R-10k blends which would not lead to reliable results; hence, it was left for a future study.

## 4. Conclusions

A detailed analysis of MD data for the orientational dynamics of ring molecules in blends with equivalent linear chains has revealed strong heterogeneities in their orientational relaxation, which become more pronounced as their molecular length or the concentration of the blend in linear chains increases. Our conclusions are based on a detailed statistical analysis of a large number of simulation data for the relaxation of individual ring molecules in these blends, obtained from very long simulations to ensure complete relaxation of all rings. The relaxation curve computed for each ring molecule in the blend was fitted to a KWW function, from which we next obtained the values of: (a) the stretching exponent *β*, (b) the characteristic KWW relaxation time $\tau_{KWW}$, and (c) the total relaxational time $\tau_{0}$. According to our analysis, the value of *β* is systematically smaller in the blends than in the corresponding pure ring melt; it also decreases with increasing concentration of the linear component, but approaches a constant value at around *φ*_L_ = 0.7. The overall relaxation time $\tau_{0}$, on the other hand, is larger in the blends, and was found to increase rapidly with increasing molecular length. 

The statistical analysis of the ensemble of relaxation times revealed a broader distribution in the blends than in the pure ring melts, which is another indication of enhanced dynamic heterogeneity. For a given molecular weight (the same between ring and linear chains), the median of the distribution shifts to higher relaxation times as the relative fraction of linear chains increases, and the distributions themselves extend out to longer times.

We attribute the enhancement of dynamic heterogeneity in the blends to strong threading events between rings and linear chains, especially to multiple threadings. With increasing size of the rings and/or increasing concentration of the blend in linear chains, rings become multiply threaded by linears which dramatically slows down their orientational relaxation and, of course, diffusion, since these topological constraints are long-lived and require the concerted release of several pairs of mutual interactions before the ring molecule can escape, and thus relax.

We further analyzed the MD data for the ring diameter TACF 〈u(t)⋅u(0)〉 in terms of a function that is the sum of simple exponential functions. For the pure R-2k and R-5k ring melts, an adequate description of the data was achieved by only three such functions whereas for their blends we needed four such functions to accurately describe the MD data. We speculated that in both cases, the first two faster relaxation modes correspond to the fast loop dynamics and slower loop migration mechanism, respectively [20,30]. The third mode, on the other hand, corresponds to the translational (or overall diffusive) motion of the center-of-mass of the ring, while the fourth (in the case of the blends), which is also the slowest in all cases, to the release of ring-linear threading events. The corresponding relaxation time *τ*_4_ and pre-exponential factor *A*_4_ were further found to depend on the fraction *φ*_L_ of linear chains in the blend.

Our analysis reveals that by just reporting the average relaxation time is not enough to describe the wealth and richness of dynamic phenomena underlying ring relaxation in their blends with linear chains. A much more enlightening and accurate picture emerges by the precise identification of the subpopulation of rings involved in threadings with linear chains, especially the subpopulation that is engaged in multiple threading events. Multiply-threaded rings exhibit the slowest dynamics and their relative subpopulation in the blend dictates the overall extent of dynamic heterogeneity. The larger this subpopulation in comparison to the rest of the rings (those that are completely un-threaded or just singly threaded), the higher the degree of dynamic heterogeneity exhibited in comparison to that in the corresponding pure ring melt.

## Figures and Tables

**Table 1:** Some technical details (molecular weight of ring and linear chains, molar fraction of linear chains, number of linear chains in the simulation cell, number of rings in the simulation cell) concerning the ring-linear PEO blends studied.

System	*M*_w_ (g/mol)	*φ*_L_	Number of Linear Chains	Number of Ring Molecules
PEO-2k	1846	0.2	30	120
		0.5	75	75
		0.7	105	45
PEO-5k	5326	0.2	38	150
		0.5	80	80
		0.7	117	50

**Table 2:** Molecular dynamics (MD) predictions for the values of the characteristic stretching exponent *β*, KWW relaxation time *τ*_KWW_, and total correlation time *τ*_0_ quantifying terminal relaxation of rings in the simulated blends. For direct comparison, we also report the corresponding data for rings in their own melt from Ref. [25].

System	*φ*_L_	*β*	*τ*_KWW_ (ns)	*τ*_0_ (ns)
PEO-2k	0	0.78 ± 0.01	7.1 ± 0.05	8.2 ± 0.1
	0.2	0.71 ± 0.01	7.6 ± 0.05	9.5 ± 0.1
	0.5	0.70 ± 0.01	8.9 ± 0.06	11.4 ± 0.1
	0.7	0.70 ± 0.01	10.2 ± 0.06	13.0 ± 0.1
PEO-5k	0	0.66 ± 0.01	50 ± 0.5	67 ± 0.5
	0.2	0.58 ± 0.01	61 ± 0.5	97 ± 0.8
	0.5	0.51 ± 0.01	91 ± 0.7	174 ± 1.5
	0.7	0.51 ± 0.01	112 ± 0.8	210 ± 2.0

**Table 3:** MD predictions for the values of the set of characteristic relaxation times *τ*_1_, *τ*_2_, *τ*_3_ and *τ*_4_ and pre-exponential constants *A*_1_, *A*_2_, *A*_3_ and *A*_4_ quantifying ring terminal relaxation in the simulated blends. For direct comparison, we also report the corresponding values for R-2k and R-5k rings in their pure melts from the corresponding analysis of the simulation trajectories presented in Ref. [25].

System	*φ*_L_	*τ*_1_ (ns)	*A*_1_	*τ*_2_ (ns)	*A*_2_	*τ*_3_ (ns)	*A*_3_	*τ*_4_ (ns)	*A*_4_
PEO-2k	0.0	0.10 ± 0.01	0.06 ± 0.01	1.1 ± 0.1	0.17 ± 0.02	10 ± 1	0.77 ± 0.04	-	
	0.2	0.09 ± 0.01	0.05 ± 0.01	1.1 ± 0.1	0.20 ± 0.02	10 ± 1	0.60 ± 0.03	20 ± 2	0.15 ± 0.01
	0.5	0.12 ± 0.01	0.07 ± 0.01	1.3 ± 0.1	0.14 ± 0.02	9 ± 1	0.41 ± 0.02	20 ± 2	0.38 ± 0.02
	0.7	0.09 ± 0.01	0.06 ± 0.01	1.3 ± 0.1	0.15 ± 0.02	9 ± 1	0.40 ± 0.02	24 ± 1	0.39 ± 0.03
PEO-5k	0.0	1.1 ± 0.1	0.12 ± 0.01	11 ± 1	0.23 ± 0.02	95 ± 8	0.65 ± 0.04	-	-
	0.2	1.0 ± 0.1	0.10 ± 0.01	10 ± 1	0.25 ± 0.02	90 ± 8	0.49 ± 0.03	340 ± 15	0.16 ± 0.01
	0.5	1.1 ± 0.1	0.11 ± 0.01	12 ± 1	0.22 ± 0.02	93 ± 8	0.29 ± 0.02	465 ± 20	0.38 ± 0.02
	0.7	1.1 ± 0.1	0.09 ± 0.01	11 ± 1	0.23 ± 0.02	89 ± 8	0.23 ± 0.02	365 ± 15	0.45 ± 0.03
